# A novel disulfidptosis-related gene signature predicts overall survival of glioblastoma patients

**DOI:** 10.2144/fsoa-2023-0136

**Published:** 2024-05-15

**Authors:** Yuxia Zhang, Bing Liu, Yuelian Zhou

**Affiliations:** 1 Intensive Care Unit, Shandong Dongying People's Hospital, Dongying, 257091, China; 2 Department of Oncology, Shandong Dongying People's Hospital, Dongying, 257091, China; 3 Department of Social & Medical Work, Shandong Dongying People's Hospital, Dongying, 257091, China

**Keywords:** bioinformatic analysis, disulfidptosis, glioblastoma, prognosis

## Abstract

**Aim:** The aim of this study was to investigate the prognostic relevance of disulfidptosis-related genes in glioblastoma using bioinformatic analysis in The Cancer Genome Atlas Program-Glioblastoma (TCGA-GBM) database and develop a gene signature model for predicting patient prognosis. **Methods:** We conducted a bioinformatic analysis using the TCGA-GBM database and employed weighted co-expression network analysis to identify disulfidptosis-related genes. Subsequently, we developed a predictive gene signature model based on these genes to stratify glioblastoma patients into high and low-risk groups. **Results:** Patients categorized into the high-risk group based on the disulfidptosis-related gene signature exhibited a significantly reduced survival rate in comparison to those in the low-risk group. Functional analysis also revealed notable differences in the immune status between the two risk groups. **Conclusion:** In conclusion, a new disulfidptosis-related gene signature can be utilised to predict prognosis in GBM.

## Plain language summary

This research aimed to explore the importance of certain genes related to disulfidptosis in glioblastoma, a type of brain cancer. By analyzing a large database of cancer information, we identified these genes and created a model to predict how well patients with glioblastoma might do. The results showed that patients in the high-risk group, as determined by the disulfidptosis-related gene model, had a worse chance of survival compared with those in the low-risk group. This suggests that these genes could help doctors predict how glioblastoma patients will fare, which is important for their treatment and care.

## Abstract (plain language summary)

The study aimed to investigate the prognostic relevance of disulfidptosis-related genes in glioblastoma using bioinformatic analysis in the TCGA-GBM database.

Weighted co-expression network analysis was employed to identify disulfidptosis-related genes, and a gene signature model was developed to predict patient prognosis.

Patients classified into the high-risk group based on the disulfidptosis-related gene signature experienced significantly reduced survival rates compared with those in the low-risk group.

Functional analysis indicated noteworthy differences in the immune status between the two risk groups.

The findings suggest that the newly developed disulfidptosis-related gene signature can be used to predict prognosis in glioblastoma.

GBM is the most prevalent primary brain tumor and is linked to the highest mortality rate [1]. The current standard of GBM care is radiotherapy along with the chemotherapeutic drug temozolomide, although the relapse-free period is only temporary (averaging 15 months) [2]. Despite numerous studies and advances in modern medicine over the past few decades, GBM remains challenging to treat with a very poor prognosis for patients. This is mostly because we still know very little about the mechanisms behind the tumor aggressiveness of GBM.

Disulfidptosis is a newly discovered form of programmed cell death that holds promise as an anticancer strategy. Research has shown that excessive accumulation of intracellular cystine leads to disulfide stress, which can trigger rapid cell death through disulfidptosis [3]. Cysteine is necessary to promote cancer cell proliferation and survival [4]. The transport of cystine is facilitated by the protein encoded by the *SLC7A11* gene [5]. So far, there is growing evidence indicating that over-expression of SLC7A11 is a hallmark of various cancer aspects, such as tumorigenesis, proliferation, metastasis, prognosis and resistance to chemotherapy [6]. Glucose-deficient cancer cells with high expression of SLC7A11 exhibit disulfide stress due to the accumulation of disulfide material, which disrupts the normal binding of disulfide bonds between cytoskeletal proteins. This leads to the collapse of the histone skeleton and cell death [3], indicating that SLC7A11-regulated disulfidptosis has the potential to be a new target for cancer therapy. However, the biofunctions of SLC7A11 in GBM have not yet been studied.

The TCGA database is a landmark cancer genomics program that has transformed our understanding of cancer and how it can be diagnosed, treated, and prevented. By exploring gene signatures in the TCGA database, researchers can gain new insights into the molecular mechanisms and heterogeneity of cancer, as well as identify novel biomarkers and therapeutic targets for personalized medicine. Recent studies demonstrated that ALDH2 and *SFXN* genes were differentially expressed in lung adenocarcinoma compared with normal tissues, associated with worse survival outcomes in the TCGA-LUAD database [7,8].

In this study, we developed a gene signature based on SLC7A11-disulfidptosis patterns to forecast prognosis and provide therapeutic care for GBM. Four disulfidptosis-related genes were associated with the survival and immune infiltration of GBM patients. The risk scores were based on differentially expressed genes (DEGs) and were found to accurately predict clinical prognosis, immune infiltration, tumor mutation compliance, and immunotherapy response. Our study successfully demonstrated the role of the disulfidptosis-associated gene signature in predicting prognosis, immune infiltration, and immunotherapy response in GBM. Our findings provide new ideas and targets for clinical immunotherapy planning and patient management.

## Materials & methods

### Microarray data

The Cancer Genome Atlas (TCGA) database was used to obtain the fragments per kilobase per million mapped reads (FPKM) standardized data of GBM. The TCGA-GBM database is a valuable and reliable source of molecular data for cancer research, as it has been extensively used and validated by many studies [9–11]. We excluded tumor samples that did not have enough clinical information for further analysis. The present study included a total of 161 GBMs with complete survival data and essential clinical variables (age, tumor, gender, grade and stage), along with five normal samples. Normalization of TCGA-GBM data, including library-size normalization and log transformation, was performed using the R package DESeq2. DESeq2 to account for the variability and uncertainty of the count data, and to provide more accurate and robust results [12,13].

### Infiltration levels of 24 immune cells in GBM samples

We quantified the infiltration abundances of 24 immune cells in GBM subjects by Cell type identification by estimating relative subsets of known RNA transcripts (CIBERSORT) tool [14–16]. Next, we merged the infiltration levels with the survival data of the GBM samples and performed univariate Cox regression analysis and Kaplan–Meier (KM) survival analysis to identify the immune cells that had shared prognostic value. We illustrated the results of the survival analyses using KM curves.

### Weighted correlation network analysis

The transcriptome data of TCGA-GBM samples were incorporated into weighted correlation network analysis (WGCNA) analysis using the R package ‘WGCNA’. We constructed a sample dendrogram with a phenotype heatmap and then calculated the optimal soft-thresholding using the soft threshold function. The dynamic tree-cutting method was used to identify different modules. Genes with similar expressions were then clustered into co-expression modules. We calculated p-values and correlation coefficients to identify the association between a co-expression module and the phenotype. To calculate the scale-free topology fitting index r2 that corresponded to different soft-thresholding parameter β values, functional *pickSoftThreshold* was used, and the β value was selected if r2 reached 0.9. The soft-thresholding power β value was then set to ten. The module with the highest correlation with differential immune cells was extracted (|correlation coefficient| >0.5, p < 0.05), in which genes with |MM| >0.8 and |GS| >0.4 were identified as key module genes. The association between the gene and the module was represented by MM, while the correlation between the gene and the characteristic was represented by GS.

### Development of a prognostic signature

We developed a prognostic signature using Least Absolute Shrinkage and Selector Operation (LASSO) analysis and multivariate Cox regression analysis. When performing lasso regression, we not only build the model, but also use cv.glmnet to perform cross-validation, to prevent the occurrence of overfitting results as much as possible. The GBM samples in the training cohort were then divided into high- and low-risk groups based on the median value of the risk score. We assessed the prognostic ability and performance of the signature by producing KM survival curves using the log-rank test and time-dependent receiver operating characteristic (ROC) curve. We further verified the stability and reliability of the signature in the internal and external validation groups using the same methods. When collecting external data, we ensure that they are of the same disease state, and try to ensure that their gene expression levels are basically consistent, and their survival measurement units are also consistent, which ensures the comparability of external data. We assumed the independent prognostic ability of the signature using univariate and multivariate Cox regression analysis. We also employed stratified analysis to assess the performance of the signature in the same clinicopathological characteristics.

### Statistical analysis

All analyses were conducted using the R programming language, and the data from different groups were compared by the Wilcoxon test. If not specified above, a p-value less than 0.05 was considered statistically significant. When performing differential analysis, we considered the possibility of false positives, so we used the FDR method in the p.adjust function to correct the obtained p-values, and eliminated false positive data as much as possible.

## Results

### The composition of tumor-infiltrating immune cells between normal & primary GBM

**Figure 1.**
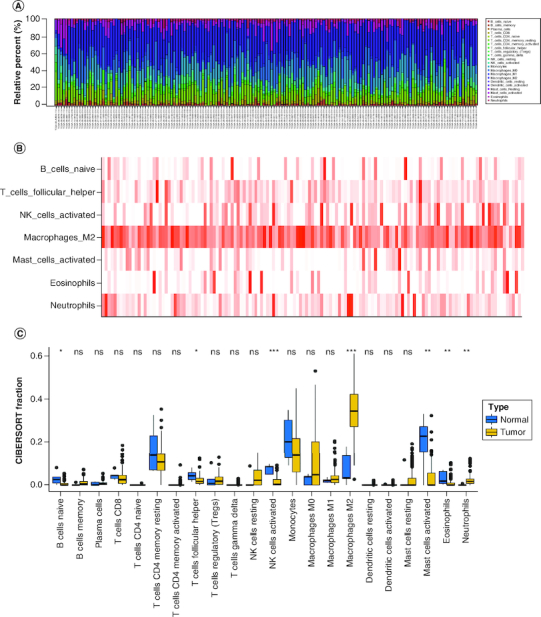
The composition of immune cells assessed by CIBERSORT algorithm in glioblastoma. **(A & B)** The identification of prominent tumor-infiltrating immune cells related to GBM. **(C)** The CIBERSORT fraction of immune cells. The blue and yellow boxes stand for the normal group and primary tumor group, respectively. CIBERSORT: Cell type identification by estimating relative subsets of RNA transcripts.

Using the CIBERSORT algorithm, we evaluated the composition of tumor-infiltrating immune cells in GBM tissue. As shown in Figure 1A & B, the results revealed that the fraction of Macrophages M2 cells was notably higher in primary tumor tissues, suggesting their potential roles in GBM. Conversely, the fraction of B cells naive, T cells follicular helper, NK cells activated, mast cells resting, eosinophils and neutrophils were significantly lower in primary GBM tumors compared with normal tissues (Figure 1C). Additionally, the sample dendrogram and trait heatmap displayed significant variation in the fractions of macrophages M2 cells (p = 0.016) between normal and primary tumors, as illustrated in Figure 1C.

### Differential immune cells-related genes by WGCNA

**Figure 2.**
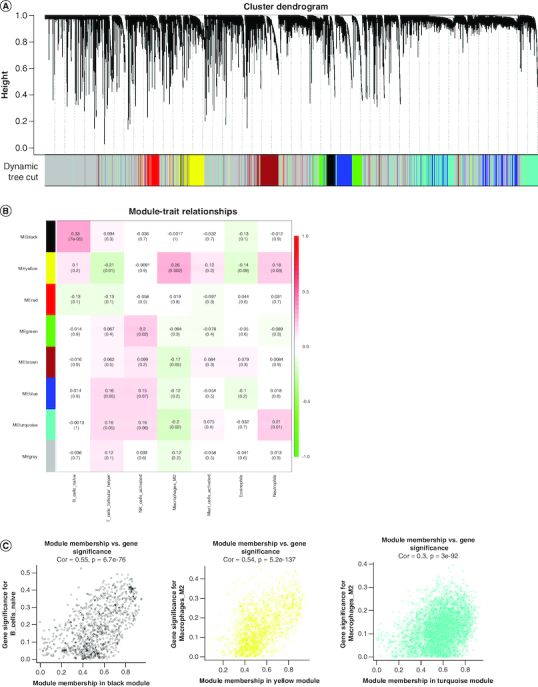
Weighted gene co-expression network analysis based on differential immune infiltration cells. **(A)** Cluster dendrogram. Genes are divided into various modules by hierarchical clustering, and different colors represent different modules, among which gray defaults to genes that cannot be classified into any module. **(B)** The heatmap of modules and immune cells correlation. In the figure, red represents a positive correlation and cyan represents a negative correlation. The darker color indicates a stronger correlation. Each frame labels the correlation coefficient, with the corresponding p-value in brackets. **(C)** The scatter plots show the correlation between module membership (x-axis) and gene signature (y-axis) in B cells and macrophages M2, related to all three types of immune cells were obtained by taking the intersection.

We conducted WGCNA to identify genes associated with differential immune cells using genes from normal and GBM samples and differential immune cells as traits. The resulting cluster dendrogram was subjected to dynamic tree-cutting, and eight modules were developed and merged (Figure 2A). Correlations between the modules and differential immune cells were evaluated to identify the most significant correlations (Figure 2B) and based on the criteria |cor| >0.5 and p-value <0.05, we selected the yellow module as the key module, which was found to be significantly correlated with Macrophages M2. In the yellow module, genes with strong correlations with Macrophages M2 (|MM| >0.8) and high gene significance (|GS| >0.4) were considered as key module genes. A total of 127 key module genes related to Macrophages M2, 145 key module genes related to NK cells activated, and 142 key module genes related to T cells follicular helper were identified (Figure 2C).

### Develop a signature in the training cohort

**Figure 3.**
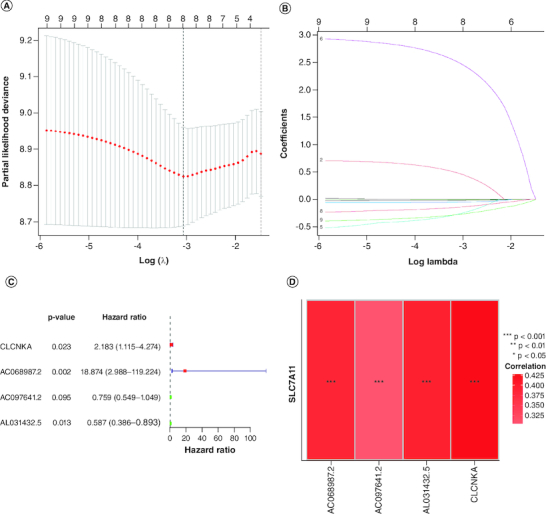
Construction of the immune-related genes prognostic signature for GBM patients. **(A & B)** Construction LASSO regression model based on SLC7A11-related immune-related genes in the training cohort. **(C)** Univariate Cox regression analysis of immune-related genes in GBM. **(D)** Correlation analysis between SLC7A11 and prognostic gene signature.

**Figure 4.**
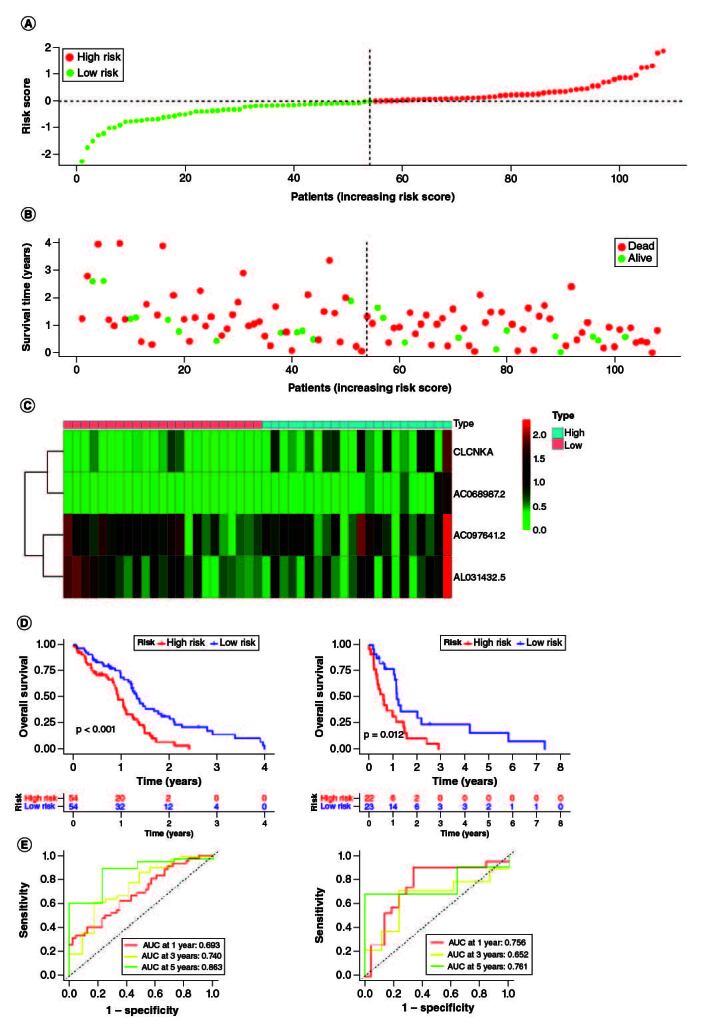
Evaluation of the prognosis prediction power of the four gene signature in the training group. **(A)** Distribution of risk score. **(B)** Survival status of GBM patients with high and low-risk scores. **(C)** The expression profiles of the four gene signatures. **(D)** Overall survival analysis of the high- and low-risk groups. **(E)** Time-dependent ROC curve of the signature.

A prognostic signature was generated using four signature genes: CLCNKA, AC068987.2, AC097641.2 and AL031432.5 (Figure 3A–D). Using the median risk score of 1.018, 161 GBM samples from the training cohort were classified into high- and low-risk groups (Figure 4A). The distribution of risk scores, survival status, and expression patterns of the four genes between the two groups are shown in Figure 4B & C. The prognostic ability of the signature was evaluated using KM survival analysis, which revealed that patients in the high-risk group had a poorer prognosis compared with those in the low-risk group (Figure 4D). Time-dependent ROC curves were generated to estimate the signature's performance, which showed that the AUC for 1-, 3- and 5-year survival prediction was 0.693, 0.740 and 0.863, respectively (Figure 4E).

### Identification of the mutation profile features of genes from each cluster

**Figure 5.**
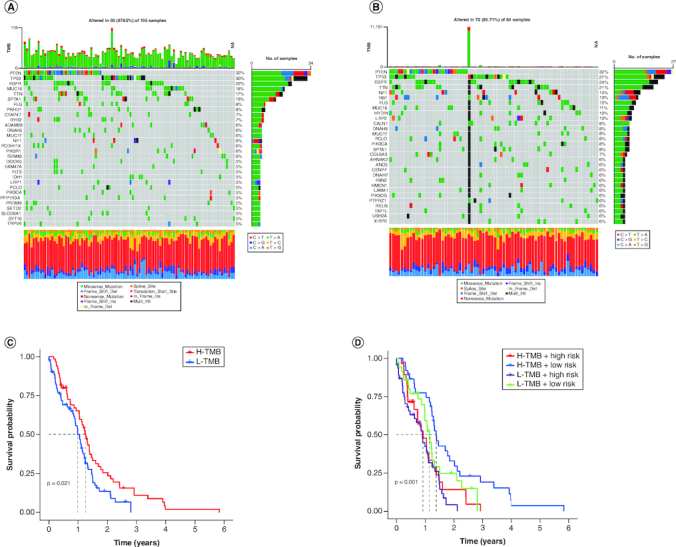
Validation of the correlation with TMB and risk score of disulfidptosis-related gene signatures in GBM patients. **(A & B)** The waterfall plot shows the mutation landscape of the top genes in GBM patients with low-risk scores and high-risk scores. **(C & D)** Kaplan–Meier survival curves revealed the prognostic value of TMB with or without combination with the risk score in GBM patients.

Accumulation of somatic DNA mutations is a known cause of cancer development. High tumor mutation burden (TMB) results in the generation of more novel antigens, rendering tumors more sensitive to immunotherapy. To determine the TMB values, we retrieved mutation data for each cluster from the TCGA database and calculated their respective TMB values. The alteration landscapes of the four clusters are shown in Figure 5A & B. Somatic mutations were compared between the two groups, and the top ten most frequently mutated genes were *PTEN*, *TP53*, *EGFR*, *TTN*, *NF1*, *RB1*, *FLG*, *MUC16*, *HYDIN* and *LRP2*. The high-risk group showed a higher frequency of TP53 mutations. Patients with high-risk scores and low TMB had the poorest prognosis among the four groups (Figure 5C & D).

### Immune infiltration analysis

**Figure 6.**
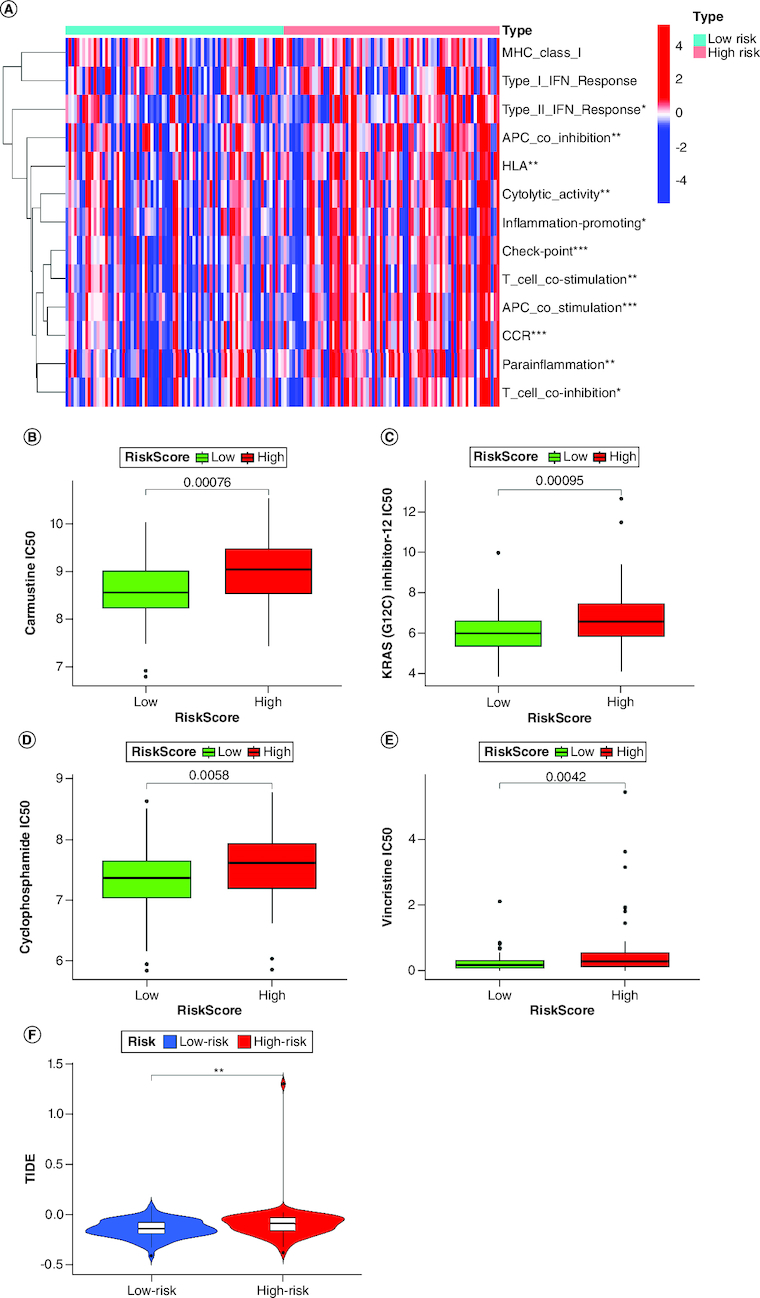
Characterization of immune infiltration in high- and low-risk GBM patients. **(A)** Correlation analysis among each immune cell proportions in GBM. **(B–E)** Sensitivity analysis between the high-risk and low-risk groups for four drugs: carmustine, KRAS(G12C), cyclophosphamidec, and vincristine. **(F)** Tumor immune dysfunction and exclusion (TIDE) analysis between the high-risk and low-risk groups.

The heat map of immune cells and immune functions comparing high-risk and low-risk groups is displayed in Figure 6A, revealing immune-cell infiltration and immune function enrichment. The results showed that several immune functions, including check-point (p < 0.001), T cell co-stimulation (p < 0.001), APC co-stimulation (p < 0.001), antigen-presenting cell (APC) co-inhibition (p < 0.01), human leukocyte antigen (HLA) (p < 0.01), cytolytic activity (p < 0.01), para-inflammation (p < 0.01), type I interferon (IFN) response (p < 0.05), T cell co-inhibition (p < 0.05), and inflammation-promoting (p < 0.05), were significantly different between the two groups. Moreover, the high-risk group exhibited better responses to carmustine, KRAS(G12C), cyclophosphamide, and vincristine, which are currently used drugs in clinics for brain cancers (Figure 6B–E). Tumor immune dysfunction and exclusion (TIDE) analysis showed that the high-risk patient group has a higher probability of immune dysfunction than the low-risk groups (Figure 6F).

## Discussion

The induction of cancer cell death is a highly effective strategy for anticancer therapy. Recent studies have highlighted the importance of disulfidptosis-induced cell death in tumorigenesis and cancer treatment. However, the role of disulfidptosis-induced cell death in GBM remains unclear. In this study, we investigated the expression of four disulfiram-related gene signatures in GBM and their association with overall survival. We developed and validated a novel prognostic model that integrates the expression of these four genes. We calculated a risk score, which accurately predicts patient prognosis and response to immunotherapy, and can serve as a new biomarker for prognosis and treatment.

Immunotherapy is an important method to prevent and treat tumors [17]. Currently, there is a lack of studies on GBM screening new prognostic biomarkers related to the immune microenvironment. Thus, many researchers are working on this area. In this study, the goal was to identify prognostic biomarkers for GBM. Our findings indicated that the disulfidptosis pathway is associated with the infiltration of multiple immune cells in GBM. The high and low-risk groups in TCGA-GBM have different fractions of antigen-presenting cell and T cell. Previous studies have demonstrated that dysfunctional antigen-presenting cells and T cells are related to poor prognosis in GBM patients due to their role in immune invasion [18]. Moreover, high-risk groups correlated with impaired antitumor immunity, including human leukocyte antigen, cytolytic activity, para-inflammation, type I interferon response, and inflammation-promoting. Therefore, attenuated antitumor immunity in patients with high risk may be an explanation for their poor prognosis. APCs capture and present antigens from tumor cells to T cells, which can then activate other immune cells to attack the tumor. However, glioblastoma cells can impair the function of APCs by secreting immunosuppressive factors. The current clinical approach is to use modified APCs that can stimulate T cells more effectively. For example, dendritic cells (DCs) are loaded with synthetic peptides to activate T cells with antitumor properties. T cells are the main effector cells of the adaptive immune system, and they can kill tumor cells that express specific antigens. However, glioblastoma cells can also escape from T cell recognition by altering their antigen expression. Therefore, the clinical approach is to use genetically engineered T cells that can recognize tumor-specific antigens and resist immunosuppression. For example, chimeric antigen receptor (CAR) T cells are modified to express a synthetic receptor that can bind to a specific antigen in glioblastoma. We identified APCs and T cells were dysfunctional in GBM patients, which can potentially be targeted for glioblastoma therapy.

The proposed prognostic model consisted of four disulfidptosis-related gene signatures (CLCNKA, AC068987.2, AC097641.2, AL031432.5). The disulfidptosis characteristic model has an important role in predicting prognosis, tumor immune infiltration, and tumor mutation load independently. In comparison to regular staging, the disulfidptosis characteristic risk score had a more significant predictive value for prognosis. Previously, it has been demonstrated that CLCNKA is associated with cancer stem cell characteristics of glioma [19]. Our findings suggest that CLCNKA participates in disulfidptosis, which may represent a novel therapeutic target for glioblastoma. Furthermore, previous studies have demonstrated that CLCNKA plays a role in mediating chloride channels and is also implicated in heart failure and salt-sensitive hypertension, highlighting its potential as a therapeutic target in various diseases [20]. The four disulfidptosis-related genes identified in our study include three novel long non-coding RNAs (lncRNAs) related to the immune system: AC068987.2, AC097641.2, and AL031432.5. Aberrant expression of several lncRNAs has been observed in glioblastoma, and some have been identified as oncogenic. For instance, the lncRNA MIR22HG is highly dysregulated in glioblastoma and has been implicated in tumor growth and progression [21]. Our findings suggest that the three lncRNAs, AC068987.2, AC097641.2 and AL031432.5, may have a role in regulating the development and progression of glioblastoma by modulating disulfidptosis. These lncRNAs may also have diagnostic potential as they have been shown to be differentially expressed in GBM patients compared with cancer-free individuals and may even distinguish between different types of brain tumors [22]. These gene signatures may have potential implications for the treatment decisions and patient outcomes in GBM. The previous study identified CLCNKA as a prognostic biomarker for glioma patients [19]. These genes may be inhibited or modulated by specific drugs or antibodies to reduce tumor progression and enhance survival. Further validation is needed in larger cohorts and clinical trials to confirm their prognostic and predictive value. According to our results, we suppose that these three prognostic biomarkers potentially regulate the tumorigenesis and progression of brain glioblastoma by mediating the disulfidptosis.

Studies have shown a correlation between tumor mutation burden and tumor prognosis and survival [23]. Moreover, clinical trials have indicated that immunotherapy benefits patients with high TMB [24]. Our research also found that patients with high disulfidptosis risk scores have a higher TMB and may have a longer survival period [23]. Gene mutations are common in GBM and affect various signaling pathways, such as the EGFR, phosphatidylinositol 3-kinase (PI3K), TP53, retinoblastoma (RB) and CDKN2A pathways [4]. These mutations were identified in the current study, which influences the sensitivity and resistance of GBM cells to disulfidptosis [25]. Consistent with the previous research, we found that the expression levels of these lncRNAs were correlated with the mutation status of several genes, such as EGFR, TP53, IDH1 and PTEN. Our study revealed that the disulfidptosis-related lncRNA signature could predict the prognosis and immune infiltration of GBM. However, the exact mechanisms and implications of disulfidptosis in gene mutations in GBM are still unclear and require further investigation. Furthermore, patients with a high risk of disulfidptosis may respond better to carmustine, KRAS (G12C), cyclophosphamide and vincristine. Carmustine, an alkylating agent, has been used for glioma treatment [26], and studies have shown an increase in overall survival (OS) by 2–4 months in newly diagnosed GBM patients [27]. The relationship between carmustine and disulfidptosis is not well understood. It was demonstrated that carmustine-induced disulfidptosis in glioblastoma cells by increasing the intracellular level of cystine, which is a precursor of disulfides [25]. SLC7A11 controls the process of absorbing cystine and converting it to cysteine. The study further demonstrated that carmustine enhanced the sensitivity of cancer cells to disulfidptosis by inhibiting the expression of SLC7A11, a key gene involved in disulfidptosis [25]. Glioma patients with KRAS mutations can benefit from treatment with KRAS (G12C) inhibitors [28]. Cyclophosphamide enhances glioma virotherapy by inhibiting innate immune responses [29]. Vincristine is effective in treating newly diagnosed adult and elderly patients [30]. These drugs have significant inhibitory effects on tumor cells and are a good option for patients with high disulfidptosis risk.

However, our study has certain limitations that should be acknowledged. Firstly, our research requires more independent datasets and a larger sample size to validate the accuracy of the model, despite the use of some independent datasets for validation in this study. Additionally, our study is a retrospective analysis of GBM, and further experimental studies are required to elucidate the function and mechanism of the identified genes, which will be the focus of our future investigations. Nonetheless, our study is the first to integrate the disulfidptosis subtype pattern with immune infiltration in GBM patients, providing valuable insights into the immune infiltration status of patients at different levels. In summary, our study presents a novel set of signature genes that can aid in predicting prognosis and response to immunotherapy.

## Conclusion

In conclusion, our study has shed light on the association between disulfidptosis-related genes and the prognosis of glioblastoma patients. We have developed a gene signature model that can effectively stratify patients into high- or low-risk groups, providing valuable prognostic information that can guide treatment decisions. Furthermore, our analyses of the tumor microenvironment have highlighted significant differences in immune cells and pathways between the two risk groups, suggesting potential avenues for immunotherapy in GBM. The results of our study demonstrate the immense potential of disulfidptosis as an anticancer strategy and provide a promising direction for future research.
